# Effects of Different Protein Levels on the Nitrogen Balance, Performance and Slaughtering Traits of Cinta Senese Growing Pigs

**DOI:** 10.3390/ani9121021

**Published:** 2019-11-22

**Authors:** Chiara Aquilani, Francesco Sirtori, Oreste Franci, Anna Acciaioli, Riccardo Bozzi, Doria Benvenuti, Marjeta Čandek-Potokar, Carolina Pugliese

**Affiliations:** 1 Department of Agriculture, Food, Environment and Forestry, Section of Animal Sciences, University of Firenze, 50144 Firenze, Italy; francesco.sirtori@unifi.it (F.S.); oreste.franci@unifi.it (O.F.); anna.acciaioli@unifi.it (A.A.); riccardo.bozzi@unifi.it (R.B.); doria.benvenuti@unifi.it (D.B.); carolina.pugliese@unifi.it (C.P.); 2 Agricultural institute of Slovenia, 1000 Ljubljana, Slovenia; meta.candek-potokar@kis.si

**Keywords:** carcass quality, crude protein, local breed, nitrogen emissions, protein requirement

## Abstract

**Simple Summary:**

Cinta Senese is a local Tuscan pig breed often reared extensively and characterized by slower growth rates compared to commercial pig breeds. It is thus frequent that the use of feed formulations based upon commercial breed requirements might provide too much protein for this breed. The aim of the present study was to assess protein requirements during the growing phase of Cinta Senese pigs (from 28 to 65 kg). Four dietary formulations, providing 12%, 14%, 16% and 18% of crude protein, were tested, and their effects on growth rates, lean and fat tissues deposition, carcass composition and nitrogen excretions, were evaluated in order to identify the best protein level to be adopted. Results showed that the animals which were fed the 12% diet were heavier, that they better exploited the dietary protein to increase the body mass and also that their urine contained the lowest amount of nitrogen. So, during the growing phase, feeding diets containing more than 12% of protein leads to a surplus in the protein intake which the animals are no longer able to convert into muscle. In conclusion, the diet providing 12% of protein resulted as being adequate for fulfilling Cinta Senese requirements during the growing phase. Identification of the actual protein requirements of Cinta Senese growing pigs could enhance the feeding management at the farm level, resulting thus in less nitrogen excretions as well as in reduced feed costs.

**Abstract:**

Cinta Senese is characterized by slow growth rates, which implies different nutritional requirements compared to major pig breeds. Four different crude protein levels (120, 140, 160 and 180 g/kg on as-fed basis, denoted as CP12, CP14, CP16 and CP18) were tested to assess the optimal protein requirements of Cinta Senese pigs during the growing phase. The in vivo performance, slaughtering traits and nitrogen balance were evaluated using individual pens and metabolic crates. Increasing the protein level in feed lowered the average daily gain (from 0.76 to 0.71 kg/d), final weight (63.0 kg for CP12 versus 60.7 kg for CP16) and reduced the protein conversion efficiency (from 0.37 to 0.58). Also, protein conversion in lean protein linearly increased from CP12 (4.82) to CP18 (7.43), which implies a worsening in the protein utilization efficiency. The nitrogen balance showed higher loss of N through urine (from 0.68 g/d/kg metabolic weight for CP12 to 1.14 g/d/kg metabolic weight for CP18) as the dietary CP levels increased, and a decrease in the biological value (51.78 for CP12 versus 36.54 for CP16). The results indicated that the CP12 diet was adequate for fulfilling the Cinta Senese protein requirements during the growing phase.

## 1. Introduction

Cinta Senese is an Italian local pig breed that is traditionally reared in Tuscany in outdoor extensive systems. In recent years, the assessment of optimal protein intake and the protein/energy ratio for growing pigs has been of major importance for pig nutrition research, but has been limited to lean genotypes [1,2]. As with most local pig breeds, Cinta Senese displays many physiological traits typical of obese genotypes, such as a slow growth rate and a marked predisposition to fat deposition [3]. Taking into account these specific metabolic features, proper feeding strategies and ad hoc diet formulations are needed to fulfill local breed nutritional requirements and to enhance their performance [4]. Several studies were carried out on Iberian pigs, pointing out that, under ad libitum feeding, the dietary protein level for this local breed is close to 129 g crude protein (CP)/kg DM from 15 to 50 kg [5], and on average, 95 g CP/kg DM from 50 to 100 kg body weight [6]. In a meta-analysis study, the same authors also observed that the carcass components of obese genotypes follow a different pattern of relative growth in comparison with the lean genotypes [7]. In the Cinta Senese breed, two studies were carried out, testing four diets with increasing CP contents (8%, 10%, 13% and 16%) in animals from 50 to 145 kg [8,9]. Considering both the growth performance and slaughtering traits of the whole post-weaning-fattening period, the authors stated that a content of 10% CP in the diet was adequate to fulfill Cinta Senese protein requirements. However, to better understand the protein requirements of this breed, the growing and fattening phases must be studied separately. Applying feeding models based upon the performance of lean genotypes to Cinta Senese might lead to a protein surplus in feed, which cannot be used for protein synthesis, and is excreted through the urine, leading to an energetic cost for animals and an economic loss for farmers. In addition to cost effectiveness, defining the optimal balance between protein and energy intakes for Cinta Senese pigs would also have positive consequences on the environment, lowering the nitrogenous excretions of pig farms [10,11]. This is particularly important for this breed, and for local breeds in general, that are traditionally reared outdoors in systems where animal excretions are more exposed to nitrogen leaching [12,13,14]. The present study aimed to fill the gaps in Cinta Senese protein requirements during the growing phase (from 30 to 65 kg). The research was carried out investigating the effects of four diets, differing in their protein level, on diet digestibility, nitrogen balance, in vivo performance and slaughtering traits.

## 2. Materials and Methods

The tested diets contained four protein levels: 120, 140, 160 and 180 g/kg on an as-fed basis (denoted as CP12, CP14, CP16 and CP18, respectively) and had the same protein quality in terms of essential amino acid proportional intake (Supplementary Table S1). Diet formulations were made according to the Nutrient Requirements of Swine (NRC, 2012) [15], considering, as a reference, the protein and amino acid (AA) requirements of pigs of 50 kg (110 lb, 16% CP as-fed) and by modifying the other diets, taking into account the AA balance. Table 1 shows the ingredients and chemical composition for each dietary formulation. Diets were pelleted to avoid single ingredient selection by the animals. Animals were fed twice a day (at 8:00 a.m. and 4:00 p.m. in equal meal portions). Two percent bentonite was added to each formulation to increase the acid insoluble ash (AIA), which was used as an internal marker to assess the diet’s digestibility.

Two trials were carried out to assess in vivo performance and slaughtering traits and to assess digestibility and nitrogen balance. To the first purpose, twelve Cinta Senese castrated males, weighing 28 kg live weight and 135 days old on average, were divided into four dietary groups and allocated in individual pens provided with chains as environmental enrichment and automatic nipples for water, according to animal welfare requirements. The pens were divided into three blocks of four pens in the barn where the trial took place, and each block was administered a different dietary treatment. The trial was repeated twice with a total of 24 pigs (n = 6 pigs × four dietary groups). The feeding level adopted was 0.95 × ad libitum, where animals did not have the feed always available, but troughs were manually refilled thrice a day to ensure satiety. Feed was stored separately for each individual pen to calculate the feed consumption every week by the weight difference. The first and second replication lasted 49 days and 42 days, respectively; when animals reached 60–65 kg of live weight, they were slaughtered together to determine the carcass composition, tissue distribution and nitrogen content. Six additional pigs were slaughtered at the beginning of the first trial, at an average weight of 28.8 kg, in line with the initial live weights of the 24 pigs in the trial. They were used to estimate the initial carcass composition and nitrogen content of the pigs in the trial, according to the comparative slaughter method. After slaughtering, the two half-carcasses of each animal were weighed separately, cooled for 12 h, and then the right side was anatomically dissected: head, neck, shoulder, ribs, loin plus belly and ham, all including the surrounding subcutaneous fat and skin. The anatomical cuts were weighed, and each cut was divided into subcutaneous fat plus skin, intermuscular fat, lean muscle and bone. Tissues were weighed separately and sampled for analysis, whereas bone was discarded. The subcutaneous fat, intermuscular fat and lean muscle (Longissimus dorsi (LD) separately) of each cut were analyzed for proximate composition according to official methods (Association of Official Analytical Chemists (AOAC), 2012) [16]. Total carcass lean muscle, fat and bone were calculated, and the total nitrogen (N) content in the lean muscle was also obtained as the sum of the individual cuts’ composition. Moreover, the lean muscle, fat, bone and nitrogen gains of the whole trial were calculated as the difference between the final and initial compositions. This was estimated by regression of the live weight and anatomical cut data of the six piglets slaughtered at the beginning of the trial.

The second trial was aimed to evaluate the digestibility and the nitrogen balance. Eight Cinta Senese castrated males were divided into two groups of four pigs each, and they underwent eight experimental cycles in metabolic crates (60 × 130 cm). Every animal tested all the diets at different ages, according to a Latin square design (Supplementary Table S2). The trial lasted nine weeks, and the animals were five months old. They weighed 55 kg on average at the beginning of the trial and 75 kg at the end. 

The same four formulations previously described in Table 1 were tested by eight pigs that alternately occupied the four metabolic crates. Animals were fed twice a day (at 8.00 a.m. and 4.00 p.m. in equal meal portions). The daily feed allowance was 90 g/kg metabolic weight. When present, refusal was collected before the morning feed distribution to calculate the actual intake of the previous day. Crates were supplied with water nipples and a trough, and they were adapted to separately sample feces in trays and total urine in flasks containing 20 mL 8 N sulfuric acid to avoid ammonia loss [17]. Each crate period lasted five days and was preceded by a 9-day period on the barn floor for diet adaptation. The crate period consisted of a phase of adaptation (two days) and a phase of fecal sampling and total urine collection (three days) according to Zhang and Adeola [18], and in line with other total tract digestibility trials [5,19,20]. The length of the crate period was designed to address the ministerial prescription on animal welfare (Authorization n 84/2016-PR—28/01/2016). The room temperature was set at 21 °C, and the relative humidity was approximately 80%. Animals were weighed before each turn in the crate to adjust the feeding amount based on their metabolic weight. Collection of total urine and fecal samples took place at fixed times: twice a day for feces (9:00 a.m. and 4:00 p.m.) and once a day for urine (4:00 p.m.). The daily urine production of each subject was weighed, and then, a sample of the total urine was taken. Feces and urine collected on a certain day were associated with the feed intake of the previous day to calculate digestibility and nitrogen balance.

The analytical methods used for feed, fecal and urine analysis were as follows: moisture (AOAC, 2012, ref: 934.01), crude protein (AOAC, 2012, ref: 976.05), ether extract (AOAC, 2012, ref: 950.46), ash (AOAC, 2012, ref: 942.05), NDF [21], ADF and lignin contents (AOAC, 2012, ref: 973.18). The dietary formulations and feces were also analyzed for AIA. AIA was used as an undigestible internal marker to calculate the apparent total tract digestibility (ATTD) of dietary components following the method proposed by Van Keulen and Young (1977) [22]. Similarly, chemical analysis was carried out on meat and fat samples to determine the moisture (AOAC, 2012, ref: 950.46), protein (AOAC, 2012, ref: 976.05), ash (AOAC, 2012, ref: 920.153) and ether extract contents (AOAC, 2012, ref: 991.36).

Data were analyzed by the generalized linear model (GLM) procedure (SAS, 2007) [23] according to the following models:

In vivo performance and slaughtering traits:

Y_ijkl_ = µ + P_i_ + T_j_ + B_k_ + b*X_ijkl_ +E_ijkl_

Where

Y = the lth observation;

P = the fixed effect of the ith protein content (1, …, 4);

T = the fixed effect of the jth trial (1, 2);

B = the fixed effect of kth block of pens (1, ..., 3);

X = the continuous effect of the initial live weight of the pig;

E = the random error.

The animal was the experimental unit.

Digestibility and nitrogen balance:

Y_ijkl_ = µ + P_i_ + S_j_ + D_k_ + c *X_ijk_ +E_ijkl_

Where

Y = the lth observation on the jth subject;

P = the fixed effect of the ith protein content (1, ..., 4);

S = the random effect of the jth subject;

D = the fixed effect of the kth day of sampling (1, ..., 3);

X = the continuous effect of the metabolic weight of the pig at entry into the crate;

E = the random error.

The animal was the experimental unit. Data are presented as the mean and root mean squared error (RMSE), and a *P*-value of < 0.05 was considered significant. Statistical differences among mean values were assessed by using Duncan’s multiple-range test, with the level of significance established *P*-value of < 0.05.

## 3. Results

### 3.1. In Vivo Performance and Slaughtering Traits

The dietary CP level affected the in vivo performance of Cinta Senese growing pigs. Most of the examined traits showed a significant linear response pattern to increasing CP levels in the diet, whereas quadratic regression was never significant (Table 2). The diet significantly affected the final weight (*P* = 0.050), even if the initial weight was similar. Animals fed the CP12 treatment were heavier than the CP16 animals. Additionally, the total feed intake (*P* = 0.045) and protein conversion index (*P* < 0.001) were similarly affected by diet, with CP12 animals showing higher total feed intake than CP18 animals, and showing the lowest protein conversion index (PCI). In contrast, the feed conversion index (FCI) was not affected by dietary treatment (*P* = 0.334). Overall, significant linear trends can be seen from CP12 to CP18 for all the examined traits, except, again, for FCI (*P* = 0.165). Increasing levels of dietary CP corresponded to a sharp and marked linear decrease in total feed intake (*P* = 0.006) and ADG (*P* = 0.014), while PCI markedly increased from CP12 to CP18 (*P <* 0.001).

After slaughtering, CP12 animals were heavier than CP16 and CP18 animals (*P* < 0.005) (Table 3). The dressing percentage and carcass composition did not vary among the four dietary groups, except for a linear increasing trend for ham percentage (*P* = 0.020); it is worth noting that the kidneys’ percentage of the carcass followed a linear increase from CP12 to CP18 (*P* = 0.011), with a clear separation between CP12 animals and CP16 and CP18 animals.

No significant differences among dietary groups were observed for tissue composition (Table 3) or for lean, fat or bone gain (*P* = 0.120, *P* = 0.080 and *P* = 0.344, respectively) (Table 4). However, lean and fat daily gains followed a linear decreasing trend (*P* = 0.048 and *P* = 0.012, respectively) as the dietary protein increased. Similarly, the conversion rate of dietary protein in lean protein linearly decreased from CP12 to CP18 (*P* < 0.0001), whereas feed conversion in lean was not affected by protein level (*P* = 0.397).

### 3.2. Digestibility and Nitrogen Balance

Table 5 shows the feed intake and total tract apparent digestibility (ATTD) of the tested dietary formulations. Dry matter intake was the same for the animals subjected to all four diets (*P* = 0.153), with a slight negative decrease (*P* = 0.034) from CP12 to CP18, already observed for animals tested in the individual pens. The CP intake increased from the CP12 to CP18 diets (*P* < 0.001), according to the experimental design. Component digestibility did not differ among the experimental diets, except for CP digestibility, which was lower in CP12 than in the other diets. CP ATTD was calculated as 1—(CP in feces CP contained in feed) and since CP contained in CP12 is the lowest, this might have affected the ratio. Moreover, the feces also contained endogenous N, which was constant for the animals subjected to all four diets, and it had a greater impact in computing the CP ATTD of CP12 (but also of CP14).

Moreover, statistical analysis also outlined a significant linear trend for CP ATTD that decreased as CP increased. At increasing levels of CP, the ATTD of dry matter followed a negative trend, outlined by the significance of linear regression (*P* = 0.032), while the digestibility of CP increased (*P* = 0.005). Table 6 shows the nitrogen balance of the four diets, and all the examined parameters were affected by the CP level. Nitrogen intake increased from CP12 to CP18 (*P* < 0.001), as did fecal N (*P* = 0.001), adsorbed N, urinary N and total excreted N (*P* < 0.001), all showing the highest levels in pigs fed the CP18 diet, and the lowest in pigs fed the CP12 diet. The abovementioned parameters followed a sharp, clear positive trend (*P* < 0.001) from CP12 to CP18; moreover, a marked separation between the CP12–CP14 and CP16–CP18 diets was easily identifiable in nitrogen excretions, both fecal and urinary. It is worth noting that for urinary N, CP12 showed the lowest value even when compared to that of CP14. Retained N was lower for the CP12 and CP16 diets than for the CP18 formulation, but it did not follow a clear trend among diets. Additionally, neither linear (*P* = 0.063) or quadratic (*P* = 0.222) trends were observed. Eventually, the biological value and the retained N/intake N ratio linearly decreased from CP12 to CP18 (*P* < 0.0001 and *P* = 0.0004), indicating a decrease in the nutritional value of the diets. Even if the diets were formulated to be isoenergetic (see Table 1), providing the same amount of gross energy, the CP level influenced some of the energy parameters. Despite the increasing fecal N, the digestible energy (DE) was unaffected by diet (*P* = 0.267). In contrast, the metabolizable energy (ME) (*P* = 0.028) linearly decreased from CP12 to CP18 (*P* = 0.044).

## 4. Discussion

Several studies have noted the physiological differences between lean and local obese pig genotypes. The slower growth rate and lower predisposition to lean tissue deposition of the latter have been widely assessed for local pig breeds, suggesting the need for specific diet formulations for each phase of growth [24,25]. Currently, this knowledge is available only for Iberian pigs whose protein and energy requirements, as well as their optimal ratio, have been studied for each rearing phase [5,6,25,26]. Previous studies on Cinta Senese evaluated the whole growing–fattening period [8,9,27]. These studies highlighted the lower protein requirements of Cinta Senese compared to that of lean genotypes, whose recommended protein intake for growing pigs is 16% CP [15]. The authors indicated that 10% CP was the optimal dietary protein level for the growing–fattening period (from 46 to 150 kg) for Cinta Senese, which is slightly lower than what was observed in the present study. Indeed, protein requirements in growing pigs are higher than during the fattening period, being mainly related to protein deposition processes, and to a lesser extent, to protein maintenance [28]. So, considering both that the target of the present study was to investigate only the growing phase and that, even for the fattening phase, the use of a protein content in the diet lower than 10% had negative effects [8], in the present research, the lowest CP level was determined to be CP12. Lowering the dietary CP content alone might lead to an AA shortage or to an uneven intake of some AAs. When an AA is first limited, protein synthesis stops, and the AAs in surplus are excreted, increasing N excretions. In the present work, all of the tested diets met the animals’ requirements for essential AAs, even if at different levels of dietary CP. Without limiting factors, protein deposition follows a linear response according to the amount of protein supplied. Once it reaches a breaking point, the protein deposition largely depends upon the energy supply [5]. The differences observed in this study among different CP levels were neither due to unbalanced AA supplementation, nor to a shortage in energy supply, as the ME was always higher than the recommended 15.70 MJ/kg dm for growing pigs [15].

The overall results of the in vivo performance and slaughtering traits suggest that supplying increasing levels of dietary CP to Cinta Senese growing pigs did not have positive effects on performance (ADG, PCI, lean gain and protein conversion in lean protein); in contrast, from CP12 to CP18, the efficiency in protein utilization and N retention (Retained/intake N) worsened. In Iberian pigs from 15 to 50 kg live weight (lw), Nieto et al., (2002) [5] observed that the dietary CP content affected ADG, protein deposition and retained/intake N ratio. Near the ad libitum feeding level, the best performance of these parameters was associated with a dietary CP content of 129 g/kg dry matter, comparable to our CP12 formulation, while increasing the content of dietary CP gradually lowered the performance. These results corroborated the evidence of the metabolic differences between lean and obese genotypes; thus, Cinta Senese pigs were able to adsorb high amounts of N at the ileal level. It can be assumed that they have an adequate enzymatic profile to degrade dietary formulations up to CP18, but once AAs are adsorbed and the genetic upper limit for protein deposition is reached, AAs are used in catabolic processes. The poor efficiency of protein catabolism is widely known [1,11,28], and the negative effects of this pathway affect overall animal performance.

In lean genotypes, genetic selection has raised the predisposition to deposit lean tissue beyond the upper limit of appetite, making high-protein diets fully exploitable by animals [5]. The effects of protein on food intake have been fully studied, and it is mainly attributable to hormonal satiety responses involving cholecystokinin as well as glucagon-like peptide-1, peptide tyrosine and gastric inhibitory polypeptide [29,30,31]. In particular, the response of cholecystokinin has been found to be different between slow-growing and fast-growing pigs, with the former reaching a higher concentration of cholecystokinin in plasma sooner after feed ingestion has begun when compared to that of fast-growing pigs [32]. In addition to the appetite depression exhibited for both the individual penned animals and those in metabolic crates, the greater CP content of the CP16 and CP18 diets ensured a higher intake of CP, so the poorer in vivo performance and the lower lean and protein deposition indices observed for those pigs were mainly related to a worse protein utilization efficiency. Additionally, the increasing weight of the kidneys as the dietary CP increased suggests a higher metabolic activity in expelling ureic N, as reported also for Iberian pigs fed increasing levels of dietary CP [6,33]. These results corroborated the nitrogen balance, clearly delineating a positive response of urinary N that increased according to the dietary CP supplied. It can be concluded that the CP12 diet was adequate to fulfill Cinta Senese protein requirements during the growth phase. Providing a CP content equal to or greater than 11.5% (CP12 formulation), as recommended for major pig breeds, had no effect on carcass composition and tissue percentages. The protein and lean gain were not enhanced, but were hindered by increasing levels of dietary CP, and N excretions linearly increased. It is worth noting that this study failed to identify a minimum level of dietary CP below which growth performances are affected by the CP’s shortage. The results observed by Sirtori et al. [8] discouraged the use of diets providing less than 10% CP even in the fattening phase; for this reason, in the present research, the minimum was determined to be CP12. 

The opportunity for further lowering the dietary CP for Cinta Senese growing pigs cannot be excluded, but the linear increase in fat gain observed from CP12 to CP18 animals might indicate that we reached the lower limit of protein to energy supply below which, lacking the AAs required for protein deposition, the energy in excess is used for fat deposition. Indeed, diets excessively poor in protein enhance the genetic predisposition to fat deposition in local breeds, leading to carcasses that are extremely fat [11], with a worse slaughtering yield and a lower consumer acceptability of meat when compared with those of leaner pigs.

## 5. Conclusions

In conclusion, among the tested dietary formulations, the CP12 diet, providing 120 g/kg crude protein to growing Cinta Senese pigs, was adequate for fulfilling their protein requirements. The CP12 formulation had no negative effects on in vivo performance or quality carcass traits, but resulted in an amelioration of the nitrogen balance, lowering the nitrogen excreted through urine.

## Figures and Tables

**Table 1:** Ingredients and chemical composition of experimental diets with different crude protein (CP) levels (% on an as-fed basis).

		Diet			
		CP12	CP14	CP16	CP18
Ingredients	%				
Maize		73.5	68.0	62.9	57.4
Soybean meal (46%)		9.0	14.5	19.5	25.0
Wheat bran		10.0	10.0	10.0	10.0
Maize oil		2.0	2.0	2.0	2.0
Bentonite		2.0	2.0	2.0	2.0
Lysine HCl		0.45	0.45	0.45	0.50
Methionine		0.05	0.05	0.10	0.10
Premix ^1^		3.0	3.0	3.0	3.0
Composition	%				
Crude Protein		11.5	13.5	15.4	17.5
Ether extract		4.5	4.4	4.2	4.1
NDF		11.3	11.2	11.1	11.1
ADF		3.7	3.8	3.9	4.1
ADL		1.3	1.7	1.8	1.7
Ash		6.5	6.8	7.0	7.3
Lysine ^2^		0.87	1.02	1.15	1.29
Methionine ^2^		0.27	0.30	0.37	0.39
Gross energy ^2^	MJ/kg	16.1	16.1	16.1	16.2

^1^ Premix provided per kg of feed: Vitamin A, 13,500 IU; Vitamin D3, 1200 IU; Vitamin E, 12 mg; Vitamin K, 2.25 mg; Vitamin B1, 2.4 mg; Vitamin B2, 4.8 mg; Vitamin B6, 1.8 mg; Vitamin B12, 0.024 mg; Niacinamide, 24 mg; Calcium pantothenate, 1.35 mg; Folic acid, 0.3 mg; Choline HCl, 495 mg; Iron(II) carbonate, 90 mg; Copper(II) sulfate, 93 mg; Manganese oxide, 30 mg; KI, 1.96 mg; Na Selenite, 0.3 mg; DL Methionine, 105 mg; and L Lysine, 60 mg. ^2^ Calculated according to tabulated data of the ingredients.

**Table 2:** Effects of different dietary levels of CP on the in vivo performance of Cinta Senese growing pigs (n = 24).

	Diet				RMSE	*P*-Value				
	CP12	CP14	CP16	CP18		Diet	Trial	Initial Weight	Linear	Quadratic
Initial weight (kg)	28.32	27.81	28. 30	28.33	2.60	0.900	0.021	-	0.738	0.615
Final weight (kg)	63.04 ^a^	61.82 ^ab^	60.72 ^b^	61.01 ^ab^	1.81	0.050	0.593	0.0002	0.010	0.905
Total feed intake (kg)	113.1 ^a^	111.8 ^ab^	110.6^ab^	108.3 ^b^	2.8	0.045	<0.0001	0.406	0.006	0.545
Total CP intake (kg)	13.01 ^D^	15.12 ^C^	17.09 ^B^	19.04 ^A^	0.42	<0.0001	<0.0001	0.399	0.014	0.411
ADG (kg/d)	0.76	0.74	0.71	0.72	0.04	0.064	<0.0001	0.333	0.014	0.914
FCI	3.33	3.32	3.43	3.30	0.18	0.334	0.004	0.131	0.165	0.584
PCI	0.37 ^D^	0.44 ^C^	0.53 ^B^	0.58 ^A^	0.03	<0.0001	0.010	0.232	<0.0001	0.704

ADG = average daily gain; FCI = feed conversion index (kg feed/kg weight gain); PCI = protein conversion index (kg of protein intake/kg weight gain); ^a,b,c^ Values within a row with different superscripts differ significantly at *P* < 0.05. ^A,B,C,D^ Values within a row with different superscripts differ significantly at *P* < 0.01. RMSE = Root mean square error.

**Table 3:** Effects of different dietary levels of CP on carcass composition and tissues of Cinta Senese growing pigs (n = 24).

	Diet					*P*-Value				
	CP12	CP14	CP16	CP18	RMSE	Diet	Trial	Initial Weight	Linear	Quadratic
Carcass weight (kg)	52.61 ^A^	50.92 ^AB^	49.81 ^B^	49.30 ^B^	1.67	0.005	0.131	0.001	0.001	0.810
Dressing percentage (%)	83.30	82.42	81.62	80.81	2.24	0.304	0.021	0.900	0.067	0.895
Carcass composition (%)										
Head	8.93	8.26	8.93	9.29	0.70	0.174	0.298	0.589	0.235	0.118
Neck	9.01	8.65	8.66	8.92	0.74	0.865	0.995	0.110	0.711	0.472
Shoulder	13.66	13.74	13.31	13.68	0.56	0.599	0.056	0.107	0.803	0.446
Ribs	25.70	26.11	25.01	25.32	1.25	0.455	0.028	0.246	0.360	0.928
Loin and belly	14.88	15.24	15.50	14.50	0.87	0.305	0.0001	0.023	0.657	0.116
Ham	25.27	25.52	26.00	26.19	0.64	0.116	0.003	0.115	0.020	0.789
Kidney	0.35 ^b^	0.40 ^ab^	0.46 ^a^	0.46 ^a^	0.08	0.050	0.923	0.153	0.011	0.497
Tissues (%)										
Total lean	38.32	37.61	38.21	39.60	1.68	0.813	0.647	0.068	0.509	0.549
Total fat	45.31	45.90	45.34	43.13	2.64	0.669	0.627	0.163	0.346	0.513
Subcutaneous fat	36.20	37.01	36.31	34.50	2.42	0.680	0.952	0.167	0.372	0.413
Intermuscular fat	6.55	6.40	6.37	6.44	0.60	0.839	0.009	0.932	0.926	0.386
Total bone	14.71	14.84	14.51	15.33	1.19	0.744	0.208	0.645	0.579	0.624

^a^^,b^ Values within a row with different superscripts differ significantly at *P* < 0.05. ^A,B^ Values within a row with different superscripts differ significantly at *P* < 0.01. RMSE = Root mean square error.

**Table 4:** Effects of different dietary levels of CP on slaughtering traits and protein deposition of Cinta Senese growing pigs (n = 24 pigs of averagely 65 kg and n = 6 pigs of 28 kg).

	Diet				RMSE	*P*-Value				
	CP12	CP14	CP16	CP18		Diet	Trial	Initial Weight	Linear	Quadratic
Daily gain (g/d)										
Lean	273.3	258.2	245.8	262.8	20.8	0.120	0.0001	0.264	0.048	0.507
Fat	356.7	351.2	328.7	304.9	32.1	0.080	0.0001	0.020	0.012	0.668
Bone	82.6	80.2	70.5	80.3	12.9	0.344	0.165	0.550	0.291	0.510
Protein of lean (g/d)	59.5	56.8	54.0	57.0	5.8	0.295	0.001	0.236	0.107	0.767
Conversion rates (kg/kg)										
Feed conversion in lean	9.13	9.72	9.99	9.19	0.86	0.397	0.005	0.759	0.286	0.334
Protein conversion in lean protein	4.82 ^C^	6.03 ^B^	7.00 ^A^	7.43 ^A^	0.69	<0.0001	0.020	0.604	<0.0001	0.579

Feed conversion in lean = kg of feed intake/kg of lean tissue gain; Protein conversion in lean protein = kg of protein intake/kg of lean tissue gain. ^A,B,C^ Values within a row with different superscripts differ significantly at *P* < 0.01. RMSE = Root mean square error.

**Table 5:** Intake and apparent total tract digestibility (ATTD) of experimental diets with different CP levels (n = 8).

	Diet				RMSE	*P*-Value			
	CP12	CP14	CP16	CP18		Diet	MW	Linear	Quadratic
Intake (g/d):									
- Dry matter	1752	1673	1723	1580	196	0.153	<0.0001	0.034	0.422
- Crude protein	234 ^C^	261 ^B^	308 ^A^	322 ^A^	35	<0.0001	0.0002	<0.0001	0.361
Total tract apparent digestibility:									
- Dry matter	0.869	0.864	0.858	0.853	0.011	0.080	0.007	0.032	0.567
- Organic matter	0.890	0.895	0.890	0.885	0.024	0.124	0.0007	0.061	0.989
- Crude protein	0.860 ^b^	0.865 ^ab^	0.869 ^a^	0.873 ^a^	0.010	0.016	<0.0001	0.005	0.279
- Ether extract	0.934	0.929	0.924	0.919	0.033	0.197	0.0009	0.081	0.952
- NDF	0.750	0.742	0.734	0.726	0.042	0.370	0.091	0.101	0.557

^a,b^ Values within a row with different superscripts differ significantly at *P* < 0.05. ^A,B,C^ Values within a row with different superscripts differ significantly at *P* < 0.01. RMSE = Root mean square error.

**Table 6:** Intake, balance and efficiency of nitrogen utilization of experimental diets with different CP levels (n = 8).

	Diet				RMSE	*P*-Value			
	CP12	CP14	CP16	CP18		Diet	MW	Linear	Quadratic
N intake (g/d/kg MW ^1^)	1.60 ^D^	1.88 ^C^	2.10 ^B^	2.31 ^A^	0.24	<0.0001	0.050	<0.0001	0.661
Nitrogen balance:									
Fecal N (g/d/kg MW)	0.23 ^C^	0.25 ^BC^	0.27 ^AB^	0.30 ^A^	0.05	0.002	<0.0001	0.0004	0.548
Absorbed N (g/d/kg MW)	1.38 ^D^	1.63 ^C^	1.83 ^B^	2.01 ^A^	0.21	<0.0001	0.373	<0.0001	0.512
Urinary N (g/d/kg MW)	0.68 ^C^	0.79 ^B^	1.10 ^A^	1.14 ^A^	0.14	<0.0001	0.036	<0.0001	0.011
Total excreted N (g/d/kg MW)	0.89 ^C^	1.04 ^B^	1.41 ^A^	1.40 ^A^	0.17	<0.0001	0.840	<0.0001	0.026
Retained N (g/d/kg MW)	0.72 ^b^	0.84 ^ab^	0.70 ^b^	0.91 ^a^	0.22	0.018	0.061	0.063	0.222
Biological Value ^2^	51.78 ^A^	51.79 ^A^	36.54 ^C^	44.97 ^B^	9.68	<0.0001	0.027	0.0004	0.030
Retained/intake N	44.27 ^A^	43.73 ^A^	31.84 ^B^	39.26 ^A^	8.67	<0.0001	0.133	0.002	0.043
Digestible Energy (MJ/kg DM)	16.71	16.60	16.60	16.74	34.81	0.267	0.001	0.994	0.066
Metabolizable Energy (MJ/kg DM)	16.54 ^a^	16.36 ^ab^	16.23 ^b^	16.38 ^ab^	34.36	0.028	0.001	0.044	0.018
ME:DE	98.80	98.49	97.82	97.82	10.36	0.616	0.108	0.332	0.346

ME = metabolizable energy; DE = digestible energy. ^1^ Metabolic weight: (kg live weight) ^0.75^. ^2^ Retained/Absorbed. ^a,b^ Values within a row with different superscripts differ significantly at *P* < 0.05. ^A,B,C,D^ Values within a row with different superscripts differ significantly at *P* < 0.01. RMSE = Root mean square error.

## Supplementary Materials

The following are available online at https://www.mdpi.com/2076-2615/9/12/1021/s1, Table S1: Amino acids composition (% on fed-basis) of experimental diets in comparison to ideal protein amino acid profile (NRC, 2012) for 25 to 50 kg pigs, Table S2: Latin square design for digestibility trial in metabolic crate, Table S3: Experimental design.
