# Supplementary material for: Effects of Different Protein Levels on the Nitrogen Balance, Performance and Slaughtering Traits of Cinta Senese Growing Pigs
Source: Animals (Basel). 2019 Nov 22;9(12):1021. doi: 10.3390/ani9121021 (PMC6940795; doi:10.3390/ani9121021)
Supplement: Supplementary file 1 [file animals-09-01021-s001.pdf]

Supplementary material

Supplementary Table S1 *Amino acids composition (% on fed-basis) of experimental diets in comparison to ideal protein amino acid profile (NRC, 2012) for 25 to 50 kg pigs.*

	Ideal	CP12	CP14	CP16	CP18
Arg	0.50	0.66	0.83	0.98	1.08
His	0.39	0.33	0.39	0.44	0.49
Ile	0.59	0.45	0.55	0.64	0.70
Leu	1.13	1.11	1.26	1.39	1.49
Lys	1.12	0.87	1.02	1.15	1.29
Met	0.32	0.27	0.30	0.37	0.39
Phe	0.68	0.55	0.66	0.76	0.84
Thr	0.72	0.43	0.52	0.60	0.66
Trp	0.19	0.13	0.16	0.19	0.20
Val	0.75	0.55	0.65	0.74	0.77
Cys	0.33	0.28	0.30	0.33	0.35

Supplementary Table S2 *Latin square design for digestibility trial in metabolic crate*

	Crate CP12	Crate CP14	Crate CP16	Crate CP18
Week 1	a	b	c	d
Week 2	e	f	g	h
Week 3	b	c	d	a
Week 4	f	g	h	e
Week 5	c	d	a	b
Week 6	g	h	e	f
Week 7	d	a	b	c
Week 8	h	e	f	g

[2] *a, b, c, d, e, f, g, h* are the experimental animals

Supplementary Table S3 *Experimental design*

	Animals		Samples type	Sampling
Individual pens	24 Cinta Senese castrated males (+6 animals immediately slaughtered)<br>Total= 30 animals	*In vivo*	Weight, FDI, ADG, backfat thickness	Once a week
		Post-mortem	Carcass: weight and yield	At slaughtering
			Anatomical cuts (head, neck, shoulder, ribs, loin, ham): weight and tissue composition (subcutaneous fat plus skin, intermuscular fat, lean and bone)	12 hours after slaughtering
Metabolic cages	8 Cinta Senese castrated males	*In vivo*	Weight, FDI, ADG	Every time animal was put in metabolic cage
			Urine collection	Daily at 4.00 pm
			Fecal sampling	Twice a day at 9.00am and 4.00 pm
